# HIF1α reinforces PPARγ-dependent metabolic rechanneling to support lipid accumulation in adipocytes

**DOI:** 10.3389/fmmed.2026.1716464

**Published:** 2026-02-23

**Authors:** Chaonan Zhu, Meiqian Wu, Minh Duc Pham, Yue Wang, Arka Provo Das, Yijie Mao, Peter Mirtschink, Ting Yuan, Jaya Krishnan

**Affiliations:** 1 Department of Medicine, Cardiology, Goethe University Hospital, Frankfurt, Germany; 2 Institute for Cardiovascular Regeneration, Centre for Molecular Medicine, Goethe University Frankfurt am Main, Frankfurt am Main, Germany; 3 Genome Biologics, Frankfurt am Main, Germany; 4 Institute for Clinical Chemistry and Laboratory Medicine, Faculty of Medicine and University Hospital Carl Gustav Carus, Technische Universität Dresden, Dresden, Germany; 5 Cardiopulmonary Institute, Frankfurt, Germany; 6 DZHK (German Centre for Cardiovascular Research), Partner Site RheinMain, Frankfurt am Main, Germany

**Keywords:** adipose tissue, HIF1α, high-fat diet, metabolism, obesity

## Abstract

**Introduction:**

Adipose tissue hypoxia is a hallmark of obesity and partly contributes to metabolic dysfunction through effects on differentiated adipocytes. Although hypoxia-inducible factor 1α (HIF1α) is a key transcriptional mediator of hypoxic responses, its state-dependent metabolic role remains incompletely defined in mature adipocytes. Herein, we investigate how HIF1α regulates lipid metabolism in differentiated adipocytes under nutrient excess conditions.

**Methods:**

An adipocyte-specific Hif1α knockout mouse model was subjected to high-fat diet feeding *in vivo*. Adipose mass, adipocyte size, glucose tolerance, and insulin sensitivity were assessed. Metabolic and enzymatic analyses focused on lipid anabolic pathways, including glycolysis-linked glycerolipid biosynthesis and PPARγ-dependent programs.

**Results:**

Adipocyte-specific deletion of Hif1α attenuated adipocyte hypertrophy, resulting in reduced adipose mass as well as improved systemic glucose tolerance and insulin sensitivity during high-fat diet feeding. Mechanistically, HIF1α reinforced PPARγ-dependent lipid anabolic programs by coordinating glycolytic flux with glycerolipid biosynthesis to promote the rechanneling of glucose-derived intermediates into triacylglyceride synthesis.

**Discussion:**

Together, these findings provide metabolic and enzymatic validations of a late-stage, state-dependent HIF1α–PPARγ lipid storage program and implicate the glycerol-3-phosphate dehydrogenase 1 (GPD1)–glycerol-3-phosphate acyltransferase (GPAT) axis as a key molecular executor of hypertrophic lipid accumulation in differentiated adipocytes.

## Introduction

Obesity is a complex metabolic disorder linked with increased risk of developing common diseases, including type 2 diabetes, hypertension, dyslipidemia, cardiovascular disease, and cancer, through excessive expansion of white adipose tissue (WAT) (Montague and O’Rahilly, 2000; Schenk et al., 2008; Piché et al., 2020). Specifically, increased visceral fat mass is the best predictor of obesity-related morbidity (Montague and O’Rahilly, 2000). WAT expansion in obesity often leads to insufficient vascularization and development of local hypoxia, which have been identified as key contributors to inflammation, fibrosis, and impaired insulin sensitivity (Choe et al., 2016). Although increases in both adipocyte cell sizes and cell numbers contribute to growth of adipose tissue, the former is strongly influenced by diet. Indeed, nutrient overload is a key factor in promoting the obese state that induces adipocyte hypertrophy as well as promotes the development of insulin resistance and glucose intolerance (Chawla et al., 2011; Makki et al., 2013; Huh et al., 2014; McLaughlin et al., 2016).

WAT is a dynamic endocrine organ whose secretory profile is markedly altered during obesity, and the enlarging adipocytes often outgrow their vascular supply and lead to local hypoxia (Trayhurn and Wood, 2004; Trayhurn et al., 2008; Blüher, 2009; Rosen and Spiegelman, 2014). In obese humans and mouse models, visceral adipose tissue (VAT) is often poorly oxygenated, which is at least partly caused by the inability of preexisting adipose tissue vasculature to meet the oxygen demands of the expanding adipose mass resulting from chronic and excessive nutrient consumption (Wood et al., 2009). Hypoxia in the expanding adipose depots triggers inflammation, fibrosis, and altered adipocyte lipid handling, including impaired fatty acid uptake and increased lipolysis, which contribute to ectopic lipid deposition and systemic metabolic disturbances (Engin, 2017; Mirabelli et al., 2024; Huynh et al., 2025). Moreover, hypoxic adipose tissue dysfunction is linked with increased release of free fatty acids and impaired fatty acid uptake, which promote lipotoxicity and metabolic inflexibility in the peripheral organs (Bays et al., 2024). Parallelly, obesity-associated WAT expansion and hypoxic stress can profoundly alter adipose tissue lipid metabolism. Dysregulation of lipid handling within the adipocytes leads to accumulation and altered production of bioactive lipid species, which function as signaling molecules to modulate inflammatory responses and insulin sensitivity. Changes in adipose-derived lipid mediators are directly linked with adipose tissue inflammation and insulin resistance in obesity (Kojta et al., 2020), highlighting lipid metabolism as a key intermediary between tissue hypoxia and metabolic dysfunction. These findings emphasize that hypoxia not only drives structural remodeling of the adipose tissue but also modulates lipid metabolism to exacerbate inflammation and insulin resistance.

In mammalian cells, decreased tissue oxygenation or hypoxia is associated with the activation of hypoxia-inducible factors (HIFs), which are dimers composed of oxygen-regulated HIF1α, HIF2α, or HIF3α subunits and a constitutively expressed HIF1β (also known as ARNT) subunit, along with transcriptional induction of the HIF target genes. The products of HIF target genes mediate induction of adaptive cellular processes, including promotion of angiogenesis, regulation of cell proliferation and survival, and alterations to cellular metabolism (Gordan et al., 2007). Evidence suggests that among the HIFα subunits, the heterodimer of HIF1α and HIF1β that forms the HIF1 complex preferentially activates genes important for glycolysis, whereas the HIF2 complex favors activation of genes involved in angiogenesis (Hu et al., 2003).

Several lines of evidence suggest that HIF1 accumulates in the adipocytes of obese humans and mouse models of obesity (Halberg et al., 2009; Krishnan et al., 2012; Kihira et al., 2020; Kudo et al., 2023; Huynh et al., 2025) and that increased HIF1α protein leads to increased adipose fibrosis in obesity (Sun et al., 2013; Guglielmi et al., 2015). Functional studies of Hif1α in mouse WAT through overexpression of a constitutively active HIF1α (Halberg et al., 2009), expression as a dominant-negative form (Zhang et al., 2010; Sun et al., 2013), deletion of the gene in normotrophic WAT (Jiang et al., 2011), or inducible ablation in hypertrophic adipocytes (Krishnan et al., 2012) have revealed the important role of this transcription factor in regulating adipocyte metabolism under hypoxic and nutrient overload conditions. Recent studies on human obesity and animal models have emphasized the central role of HIF signaling in coordinating hypoxia-driven adipose tissue dysfunction. Activation of the HIF pathways in obese adipose tissue promotes inflammatory gene expression, immune cell recruitment, extracellular matrix remodeling, and metabolic reprogramming, thereby contributing to insulin resistance and systemic metabolic impairment (Mirabelli et al., 2024; Huynh et al., 2025). Importantly, accumulating evidence indicates that the metabolic actions of HIF1α in adipose tissue are highly dependent on context, varying with adipocyte differentiation state, duration of hypoxia, and degree of metabolic stress. Although sustained or early HIF1α activation has been shown to impair adipocyte differentiation, adipose tissue expandability, and tissue architecture, HIF1α signaling in mature adipocytes can promote metabolic reprogramming to facilitate lipid storage (Mirabelli et al., 2024; Huynh et al., 2025). Disentangling these distinct processes is essential for interpreting the role of HIF1α in obesity and metabolic diseases.

The development of cardiac hypertrophy is reportedly associated with HIF1α-dependent reprogramming of cellular metabolism that encompasses a switch from fatty acid utilization to glycolysis and increased synthesis of free fatty acids (FFAs) and triacylglycerides (TAGs) (Kim et al., 2006; Krishnan et al., 2009). In this context, the switch to a glycolytic form of metabolism is attributable to the activation of genes whose products encode glucose transporters, glycolytic enzymes, lactate dehydrogenase A (Ldh-A) and pyruvate dehydrogenase kinase 1 (Pdk1), which are involved in shunting pyruvate away from the mitochondria (DeBerardinis et al., 2008). However, Kudo et al. (2023) demonstrated that HIF1α inhibits early adipocyte differentiation and promotes lipid accumulation in mature peroxisome proliferator-activated receptor γ (PPARγ)-competent adipocytes, thereby resolving the discrepancies noted earlier (Kudo et al., 2023). Thus, by directly activating the glycolytic and indirectly lipid anabolic pathways, HIF1 promotes lipid accumulation in the cardiomyocytes in response to pathological stressors known to induce cardiac hypertrophy (Krishnan et al., 2009; Knutson et al., 2021; Kudo et al., 2023). Herein, we investigated the molecular mechanisms driving this late-stage metabolic switch. Thus, we specifically examined the role of HIF1α in differentiated and PPARγ-competent adipocytes under nutrient overload rather than its functions during adipogenesis or adipose tissue development.

Altered or dysfunctional metabolite channeling has been shown to regulate DNA replication, cell proliferation, and cell survival. However, it is unclear whether rechanneling of metabolites facilitates transition to obesity and how these rechanneling events are regulated in expanding adipocytes. Therefore, we aimed to define how flux along the key metabolic pathways is differentially regulated between normal and hypertrophic adipocytes. We examined whether dysregulated metabolic flux has a regulatory role in basal adiposity, leading to dysregulated metabolite buildup and subsequent rechanneling of the glycolytic intermediates into the glycerolipid shunt and *de novo* TAG synthesis in hypertrophic adipocytes.

Accordingly, all mechanistic conclusions from this study are restricted to differentiated PPARγ-competent adipocytes and should not be extrapolated to adipocyte differentiation, adipose tissue development, or adipose tissue expandability.

## Methods

### Animal breeding and maintenance

Hif1α^fl/fl^ mice were kindly provided by Prof. Randall S. Johnson (University of California, San Diego, CA, United States). The fatty acid-binding protein 4 (Fabp4/aP2-cre/+) line was generously provided by Prof. Ronald M. Evans (University of California, San Diego) (He et al., 2003). These strains were intercrossed to generate adipocyte-specific Hif1α knockout mice (aP2-Cre; Hif1α^fl/fl^, hereafter referred to as Hif1α cKO). Littermates not carrying the Cre allele (Hif1α^fl/fl^) were used as controls. Four-week-old mice were randomly assigned to normal chow diet (NCD, Harlan Teklad Rodent Diet 8604 containing 12.2%, 57.6%, and 30.2% calories from fats, carbohydrates, and proteins, respectively) or high-fat diet (HFD, Research Diets, New Brunswick, NJ, United States, containing 58%, 26%, and 16% calories from fats, carbohydrates, and proteins, respectively) feeding groups and maintained on their respective diets for 25 weeks (Krishnan et al., 2012). Plasma glucose levels were monitored under both random-fed and fasted conditions. For the random-fed measurements, blood samples were collected via tail vein puncture at the onset of the night phase. For fasting glucose measurements, the mice were deprived of food for 12 h, and blood samples were collected the following morning. The glucose levels were measured using an Accu-Chek® Aviva glucometer (Roche Diagnostics), and glycated hemoglobin (HbA1c) values were determined using a commercially available analysis kit (DCA 2000; Bayer Diagnostics). All mice used in the experiments were male and housed in a temperature-controlled room with a 12-h light/dark cycles; they were allowed free access to food and water in accordance with the guidelines of the Swiss Federal Veterinary Office (BVET).

### Glucose tolerance test (GTT) and insulin tolerance test (ITT)

The mice were fasted overnight, and blood glucose levels were determined 30 min before glucose injection. After injecting 1.0 g of glucose/kg bodyweight intraperitoneally for the GTT, the blood glucose levels were determined with an Accu-Chek Aviva glucometer at 0, 15, 30, 60, 90, 120, and 150 min using blood from the tail vein. For the ITT, after injecting 0.75 U of insulin/kg bodyweight (Actrapid, Novo Nordisk) intraperitoneally, the blood glucose levels were determined at 0, 15, 30, 60, and 90 min, as described above. The mouse data were statistically analyzed using Excel (Microsoft), and the area under the curve (AUC) values were calculated. All GTT and ITT measurements were performed on 10–15 mice of the respective genotype and diet, unless indicated otherwise.

### Immunofluorescence (IF) staining

Adipose tissue samples were collected from retroperitoneal fat depots, and IF staining was performed as described previously (Thoma et al., 2007). In brief, adipose tissues were first embedded in optimal cutting temperature medium (Sakura Finetek). Then, cryosections were prepared as described by Hirschy et al. (2006) and fixed with 4% paraformaldehyde for 10 min; next, the sections were permeabilized by incubation with 0.2% Triton X-100 for 10 min, and immunofluorescence reactions and specimen mounting were performed (Hirschy et al., 2006). The following primary antibodies were used: anti-pan-cadherin (Sigma-Aldrich, C1821) and anti-Hif1α (Santa Cruz Biotechnology, sc-13515). We obtained 4′,6-diamidino-2-phenylindole (DAPI) and phalloidin from Invitrogen. Lastly, the IF confocal images were acquired using a DMIRE2 confocal microscope (Leica) with sequential scanning.

### Immunoblotting

Mouse adipose tissues were washed twice with ice-cold phosphate-buffered saline (PBS) and lysed in SDS-PAGE sample buffer (40 mM Tris/HCl at pH 6.8, 1% SDS, 50 mM of β-ME, and 6% glycerol). Then, the protein concentrations were measured via BCA protein assay (Pierce), and the lysates were run on gradient polyacrylamide gels of 7.5%–12.5% before being transferred to nitrocellulose membranes. The membranes were blocked with 2.5% non-fat dry milk (Cell Signaling Technology) and 2.5% bovine serum albumin (Sigma) for 1 h at room temperature before overnight incubation at 4 °C with the following antibodies: anti-Hif1α (Santa Cruz Biotechnology, sc-10790), anti-Pparγ (Abcam, ab272718), anti-Gpd1 (Santa Cruz Biotechnology, sc-376219), anti-β-actin (Sigma-Aldrich, A5441), and anti-Gpat (provided by Rene Lerch of the University of Geneva, Switzerland). Immunodetection and visualization of the signals by chemiluminescence was carried out as described previously (Hirschy et al., 2006).

### Oil red O (ORO) staining

ORO staining combined with immunofluorescence was performed as described previously (Mirtschink et al., 2015), and spot detection was performed using the Anchor image analysis platform (http://www.microscopyimages.org/).

### Cell size and cell number quantification

The sizes and numbers of the adipocytes were quantified from histological sections using a standardized image analysis workflow. Fixed and stained adipose tissue sections were imaged via bright-field microscopy (Leica) equipped with a color charge-coupled device camera. The adipocyte sizes were quantified using ImageJ software on three independent WAT sections per animal showing 30–60 clearly delineated adipocytes per section. The images were spatially calibrated prior to analysis, and only intact adipocytes fully contained within the field of view were included. The number of adipocytes was determined by counting the total primary adipocytes isolated from the visceral fat pads of mice from the respective genotypes, and the counts were normalized with respect to tissue mass to ensure comparability between samples.

### Quantitative reverse transcriptase polymerase chain reaction (qRT-PCR)

The total RNA was isolated with TRIzol (Invitrogen), and cDNA was generated from individual adipose tissues using SuperScript II (Invitrogen) as per the manufacturer’s recommendation. The qPCR steps were established as recommended by the manufacturer (Roche) and analyzed on the Roche LightCycler 480 device. All experiments were performed using three mice per genotype, and the fold changes were normalized to 18S RNA levels. The qRT-PCR primers used in this work are listed in Table 1.

**TABLE 1:** Primers used in the present study.

Gene	Forward primer (5′–3′)	Reverse primer (5′–3′)
*Hif1α*	TGC​TCA​TCA​GTT​GCC​ACT​TC	CGG​CAT​CCA​GAA​GTT​TTC​TC
*Glut1*	GCA​GCA​AGA​CCG​ATG​AAC​AC	CTC​CCA​CAG​CCA​ACA​TGA​GG
*Glut4*	GGC​TCT​GAC​GTA​AGG​ATG​GG	GCC​ACG​TTG​CAT​TGT​AGC​TC
*Aldolase A1*	CCT​TAG​TCC​TTT​CGC​CTA​CCC	GAC​AGG​CGG​GTC​ATG​TTG​AA
*Vegfα*	GGA​GAT​CCT​TCG​AGG​AGC​ACT​T	GGC​GAT​TTA​GCA​GCA​GAT​ATA​AGA​A
*Pparγ*	CTG​CAG​GAG​CAG​AGC​AAA​G	GAG​CAG​AGT​CAC​TTG​GTC​ATT​C
*Gpd1*	CTG​TCA​TCG​ATC​CCG​ACT​GG	GGG​TAG​ACA​AGT​GGC​CTG​AC
*Gpat*	TCT​CCA​GCT​TCC​AGC​TAC​ACA	GGG​CTT​TGC​TTA​CTG​GTC​CTG
*18S*	GTT​CGA​CCA​TAA​ACG​ATG​CC	TGG​TGG​TGC​CCT​TCC​GTC​AAT

### Glycolytic flux measurements

Cellular glycolytic flux was measured using a Seahorse XF24 analyzer in 24-well plates at 37 °C, with corrections for positional temperature variations adjusted from four empty wells distributed evenly on the plate. The primary adipocytes were seeded at 20,000 cells per well and washed, and approximately 630 μL of non-buffered (sodium carbonate free) pH 7.4 DMEM supplemented with 10% fetal calf serum was added to each well prior to measurement. After a 15 min equilibration period, three successive 2-min measurements were performed at 5 min intervals with mixing between the measurements to homogenize the oxygen concentration in the medium; for each condition, we measured five replicates from independent wells. The glycolytic flux was then measured as described by the manufacturer. In brief, freshly prepared glucose was added to glucose-free minimal medium, and the glycolytic rate was determined by measuring the increase in extracellular acidification rate (ECAR) relative to baseline.

### Triacylglycerol content analysis

The triacylglycerol content analysis was performed as described previously (Walter et al., 2014). In brief, the adipose tissue samples were homogenized in PBS using a Potter homogenizer. The organic phase was then dried under a stream of nitrogen gas, and the extracted lipids were reconstituted in a solution of hexane/isopropanol (3:2, v/v). The TAG levels were then measured enzymatically using a TAG determination kit (Chronolab, Barcelona, Spain).

### Enzyme activity and substrate content measurements

Spectrophotometric measurements were performed using a SpectraMax 190 plate reader (Molecular Devices). After reaching steady-state conditions, the starting reagents were added and absorption changes were registered every 10 s over a duration of 20–30 min; the enzyme activities were calculated from the linear phase. For glyceraldehyde-3-phosphate dehydrogenase (Gapdh) activity measurement, the lysates were assayed in glycine (100 mM, pH 8.5), 1 mM of DTT, 0.5 mM of EDTA, 2.2 mM of glyceraldehyde-3-phosphate, and NAD^+^; the reaction was started by adding 10 mM of arsenate, and NADH formation was monitored at 340 nm and 30 °C. The glycerol-3-phosphate dehydrogenase (Gpd) activity measurements were performed with lysates assayed in glycine (100 mM, pH 8.5), 0.5 mM of EDTA, 1 mM of DTT, and 1 mM of dihydroxyacetone phosphate; the reaction was started by adding 1 mM of NADH, and NAD^+^ formation was monitored at 340 nm and 30 °C. The mitochondrial activity was assessed as described in an earlier work (Guzy et al., 2005). Here, the lysates were assayed in glycine (100 mM, pH 8.5), 0.5 mM of EDTA, 1 mM of DTT, 0.1 mM of palmitoyl-CoA, and 0.1 mM of 5,5′-dithiobis(2-nitrobenzoic acid) for glycerol-phosphate acyltransferase (Gpat) activity measurements; the reaction was started by adding NADH, and the DTNB–CoA complex formation was measured spectrophotometrically at 412 nm. The dihydroxyacetone phosphate (DHAP) levels were measured by incubating the lysates in 100 mM of glycine at pH 8.5 in the presence of 2.3 U of Gpd; the reaction was started by adding 1 mM of NADH and monitoring the absorbance changes at 340 nm. The lactate dehydrogenase (LDH) activity was measured according to the manufacturer’s instructions (Abcam, ab197000).

### Statistical analysis

All statistical analyses and tests were carried out using Microsoft Excel software. The data are represented as mean values, with the error bars indicating the standard error of the mean (±SEM). The significance was assessed by unpaired two-tailed t-test.

## Results

### Obesity drives hypoxia and Hif1α activation in visceral adipose tissue

**FIGURE 1:**
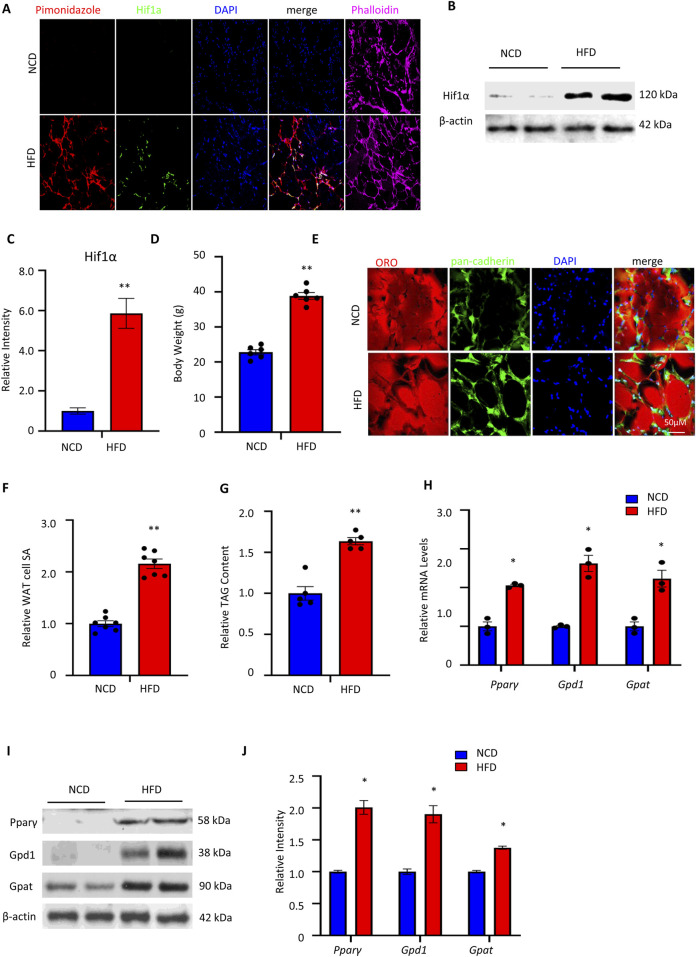
Obesity drives hypoxia and Hif1α activation in visceral WAT: **(A)** pimonidazole was injected into mice maintained on either a NCD or HFD. Visceral WAT sections of these mice were assessed for pimonidazole incorporation (red) by indirect immunofluorescence microscopy by counterstaining with anti-Hif1α antibody (green), DAPI (blue), and phalloidin to mark actin (pink). **(B, C)** Western blotting and quantitative densitometry analyses show the Hif1α protein expression levels in isolated WATs from NCD- and HFD-fed C57BL/6J mice. **(D)** Bodyweights expressed as mean ± standard error of the mean (SEM) of the NCD- and HFD-fed mice (n = 6); **p < 0.01 vs. NCD by unpaired two-tailed Student’s t-test. **(E)** WAT sections of the NCD- and HFD-fed mice were stained with the neutral lipid marker oil red O (ORO, red) and DAPI (blue) as well as counterstained with the cell boundary marker pan-cadherin (green) for immunofluorescence microscopy analysis. Scale bar: 50 μM. **(F)** Quantification of the visceral WAT cell surface areas (SAs) of NCD- and HFD-fed mice. The data are expressed as mean ± SEM (n = 7); **p < 0.01 vs. NCD by unpaired two-tailed Student’s t-test. **(G)** Quantification of the visceral WAT TAG contents of the NCD- and HFD-fed mice. The TAG content was normalized with respect to tissue weight. The data are expressed as mean ± SEM (n = 5); **p < 0.01 vs. NCD by unpaired two-tailed Student’s t-test. **(H)** Relative mRNA expression of *Pparγ*, *Gpd1*, and *Gpat* in isolated WATs from NCD- and HFD-fed C57BL/6J mice. The data are expressed as mean ± SEM (n = 3); **p < 0.01 vs. NCD by unpaired two-tailed Student’s t-test. **(I,J)** Western blotting and quantitative densitometry analyses show the Pparγ, Gpd1, and Gpat protein expression levels in isolated WATs from NCD- and HFD-fed C57BL/6J mice. NCD, normal chow diet; HFD, high-fat diet; WAT, white adipose tissue; TAG, triacylglycerol.

Nutrient overload is linked to accelerated expansion of adipose mass and the development of intra-adipose hypoxia (Schenk et al., 2008). To confirm the relationship between nutrient overload and accelerated adipose mass expansion along with Hif1α accumulation in the adipocytes, wild-type C57BL/6J mice were maintained on either NCD or HFD. The mice maintained on HFD showed increased incorporation of the hypoxia marker pimonidazole and accumulation of Hif1α protein (Figures 1A–C) along with increased bodyweights, adipose cell sizes, and TAG levels (Figures 1D–G) compared to the adipose tissues of mice fed a NCD. These data imply that adipose mass expansion in response to HFD is characterized by adipose tissue hypoxia and accumulation of Hif1α in the adipocytes, consistent with earlier results (He et al., 2011; Krishnan et al., 2012).

Adipocyte TAG levels are determinants of adipocyte size and insulin sensitivity. Pparγ is a potent inducer of TAG synthesis that has been shown to be a downstream target of HIF1α in diseased cardiomyocytes (Krishnan et al., 2009). Given the observed increases in adipocyte sizes and TAG levels of wild-type C57BL/6J mice maintained on HFD, we considered if Pparγ expression and transcriptional function could be altered in the WAT of C57BL/6J mice. Accordingly, WAT biopsies of the wild-type C57BL/6J mice subjected to the NCD and HFD protocols were assessed for the expression of Pparγ and its target genes Gpd1 and Gpat (Figures 1H–J). Gpd1 and Gpat are rate-limiting components of the glycerolipid biosynthetic shunt and are important regulatory components of *de novo* TAG synthesis (Gonzalez-Baró et al., 2007; Takeuchi and Reue, 2009). As shown in Figures 1H–J, the mRNA and protein expression of the respective genes was higher in the WAT biopsies of mice maintained on HFD compared to the controls.

### Adipocyte-specific Hif1α ablation protects from HFD-induced obesity in mouse *in vivo*

**FIGURE 2:**
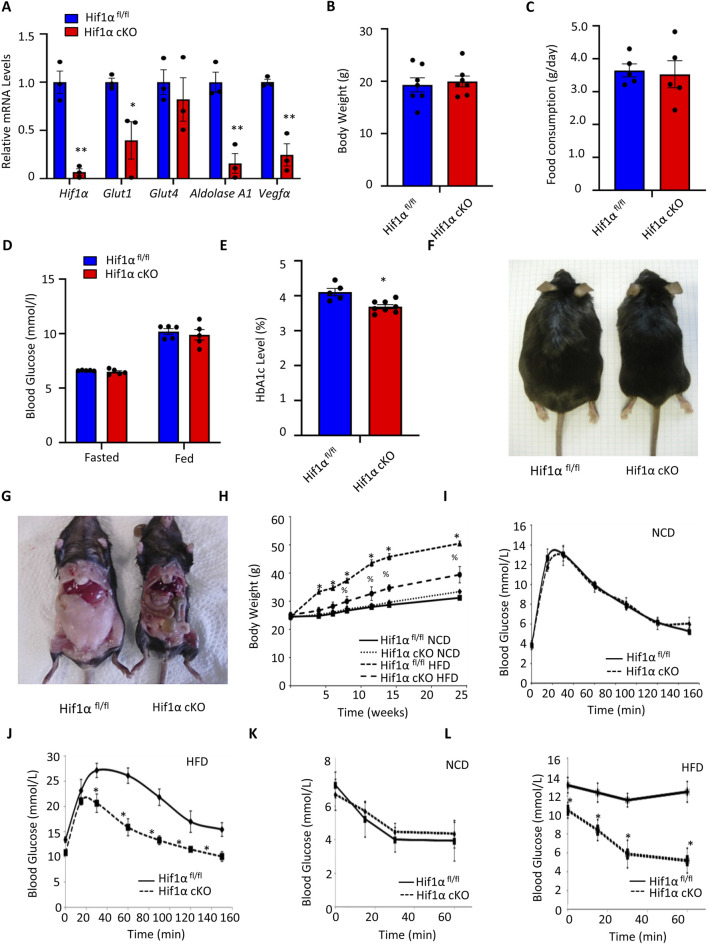
Adipocyte-specific Hif1α ablation protects against HFD-induced obesity in mouse *in vivo*: **(A)** relative mRNA expression of *Hif1α*, *Glut1*, *Glut4*, *Aldolase A1,* and *Vegf*α in HFD-fed Hif1α^fl/fl^ and Hif1α cKO mice; all values were normalized internally to 18S RNA expression and Hif1α^fl/fl^ control, respectively. The data are expressed as mean ± SEM (n = 3); *p < 0.05 vs. Hif1α^fl/fl^ by unpaired two-tailed Student’s t-test. **(B)** Bodyweights of the Hif1α^fl/fl^ and Hif1α cKO mice maintained on HFD. The data are expressed as mean ± SEM (n = 7); *p < 0.05 vs. Hif1α^fl/fl^ by unpaired two-tailed Student’s t-test. **(C)** Food intake values of the Hif1α^fl/fl^ and Hif1α cKO mice maintained on HFD. The data are expressed as mean ± SEM (Hif1α^fl/fl^: n = 5; Hif1α cKO: n = 5); *p < 0.05 vs. Hif1α^fl/fl^ by unpaired two-tailed Student’s t-test. **(D)** Fasting and random-fed blood glucose measurements in the Hif1α^fl/fl^ and Hif1α cKO mice maintained on the HFD protocol. The data are expressed as mean ± SEM (Hif1α^fl/fl^: n = 5, Hif1α cKO: n = 5); *p < 0.05 vs. Hif1α^fl/fl^ by unpaired two-tailed Student’s t-test. **(E)** Glycated hemoglobin (HbA1c) levels in the blood were measured in the Hif1α^fl/fl^ and Hif1α cKO mice maintained on the HFD protocol. The data are expressed as mean ± SEM (Hif1α^fl/fl^: n = 5; Hif1α cKO: n = 8); *p < 0.05 vs. Hif1α^fl/fl^ by unpaired two-tailed Student’s t-test. **(F, G)** Representative images of the Hif1α^fl/fl^ and Hif1α cKO mice maintained on the HFD protocol. **(H–L)** Hif1α^fl/fl^ and Hif1α cKO mice were fed a NCD or HFD for 24 weeks. **(H)** Bodyweight expressed as mean ± SEM (Hif1α^fl/fl^ NCD: n = 5; Hif1α cKO NCD: n = 5; Hif1α^fl/fl^ HFD: n = 7; Hif1α cKO HFD: n = 7); *p < 0.05 vs. Hif1α^fl/fl^ NCD and ^%^ *p* < 0.05 vs. Hif1α^fl/fl^ HFD by unpaired two-tailed Student’s t-test. **(I)** Glucose tolerance test (GTT) results from Hif1α^fl/fl^ and Hif1α cKO mice maintained on the NCD protocol. The data are expressed as mean ± SEM (Hif1α^fl/fl^ NCD: n = 5; Hif1α cKO NCD: n = 5); *p < 0.05 vs. Hif1α^fl/fl^ NCD by unpaired two-tailed Student’s t-test. **(J)** GTT results from Hif1αfl/fl and Hif1α cKO mice maintained on the HFD protocol. The data are expressed as mean ± SEM (Hif1α^fl/fl^ HFD: n = 7; Hif1α cKO HFD: n = 7); *p < 0.05 vs. Hif1α^fl/fl^ HFD by unpaired two-tailed Student’s t-test. **(K)** Insulin tolerance test (ITT) results from Hif1α^fl/fl^ and Hif1α cKO mice maintained on the NCD protocol. The data are expressed as mean ± SEM (Hif1α^fl/fl^ NCD: n = 5; Hif1α cKO NCD: n = 5); *p < 0.05 vs. Hif1α^fl/fl^ NCD by unpaired two-tailed Student’s t-test. **(L)** ITT results from Hif1α^fl/fl^ and Hif1α cKO mice maintained on the HFD protocol. The data are expressed as mean ± SEM (Hif1α^fl/fl^ HFD: n = 7; Hif1α cKO HFD: n = 7); *p < 0.05 vs. Hif1α^fl/fl^ HFD by unpaired two-tailed Student’s t-test. NCD, normal chow diet; HFD, high-fat diet; WAT, white adipose tissue; BAT, brown adipose tissue.

Given prior evidence that HIF1α acts downstream of PPARγ in mature adipocytes, we examined whether HIF1α directly controls metabolic flux through the lipid biosynthetic pathways *in vivo*. To address this, we generated mice deficient for Hif1α specifically in the adipocytes. Mice carrying loxP-flanked Hif1α alleles (Hif1α^fl/fl^) (Krishnan et al., 2009) were crossed with Fabp4/aP2-Cre recombinase (Cre) transgenic mice (He et al., 2003) to obtain Hif1α cKO mice. The Fabp4/aP2-Cre transgene induces Cre-mediated recombination in both white and brown adipocytes. Deletion of Hif1α was confirmed by PCR-mediated detection of the recombined alleles and showed ∼90% downregulation of Hif1α mRNAs in the WAT via qPCR (Figure 2A). Furthermore, the target genes of Hif1α, including solute carrier family 2 facilitated glucose transporter 1 (SLC2A1/Glut1), aldolase A1, and vascular endothelial growth factor α (Vegfα), were similarly repressed while the non-Hif1α target SLC2A4/Glut4 was largely unaffected (Figure 2A). The Hif1α cKO mice were born at the expected Mendelian ratio, had comparable bodyweights to the control Hif1α^fl/fl^ mice, and did not exhibit any overt phenotype (Figure 2B). Although the food consumption as well as fasting and post-prandial blood glucose levels were similar between the Hif1α^fl/fl^ and Hif1α cKO mice (Figures 2C,D), the HbA1c value that indicates the average blood glucose level over a prolonged period of time was consistently lower in the Hif1α cKO mice compared to the Hif1α^fl/fl^ controls (Figure 2E).

To assess whether the observed increase in adipose Hif1α in the HFD-fed mice contributed to adipose tissue expansion, both Hif1α^fl/fl^ and Hif1α cKO mice were maintained on either NCD or HFD, and their weight gain was assessed over a period of 24 weeks (Figures 2F–H). Although negligible differences were detected in the Hif1α^fl/fl^ and Hif1α cKO mice maintained on NCD, there were marked decreases in weight gain and adiposity as early as 4 weeks post-HFD in the Hif1α cKO mice compared to the Hif1α^fl/fl^ mice (Figures 2F–H). Consistent with the findings of previous studies targeting mature adipocytes (Jiang et al., 2011; Krishnan et al., 2012) and the “late-stage” perturbation outcomes mapped by Kudo et al. (2023), we observed that aP2-Cre-mediated Hif1α deletion reduced the fat mass (Figures 2F,G). This pattern of reduced adiposity and bodyweight gain in the Hif1α cKO mice in response to HFD continued throughout the 24-week period of nutrient overload (Figure 2H). Despite the reduction in WAT mass, the Hif1α cKO mice did not display a lipodystrophic phenotype as evidenced by the lack of ectopic fat redistribution to peripheral tissues like the liver or skeletal muscles (Supplementary Figures S1A, B). The liver and skeletal muscles of the Hif1α cKO mice had comparable weights and gross morphologies to those of the Hif1α^fl/fl^ mice and did not show evidence of increased lipid content or morphological alterations (Supplementary Figures S1A, B).

Defective glucose homeostasis and loss of peripheral insulin sensitivity are consequences of excessive adiposity and are partly caused by ectopic deposition of fat in the liver and skeletal muscles. To determine the impacts of both NCD and HFD on glucose homeostasis upon adipocyte Hif1α inactivation, the Hif1α^fl/fl^ and Hif1α cKO mice were subjected to a GTT. As shown in Figure 2I, the NCD-fed mice of both groups responded comparably to the GTT. In contrast, within the HFD group, the Hif1α cKO mice demonstrated significantly improved glucose handling and sensitivity than the Hif1α^fl/fl^ mice (Figure 2J). As differences in glucose handling are attributed to either decreased peripheral insulin sensitivity or insulin secretion, we performed an ITT. Although peripheral insulin sensitivity was comparable between the mice of both groups under the NCD, we observed a marked difference in peripheral insulin sensitivity in the Hif1α cKO mice maintained on HFD (Figures 2K,L). Although Hif1α^fl/fl^ mice maintained on the HFD demonstrated minimal response to a bolus insulin injection, the blood glucose levels in the Hif1α cKO mice dropped significantly in response to insulin, indicating the maintenance of normal peripheral insulin sensitivity. Together, these data implicate the key role of Hif1α in nutrient-overload-induced adipose mass expansion and maintenance of peripheral insulin sensitivity.

### Adipocyte-specific Hif1α ablation reduces adipocyte cell sizes and numbers in HFD-induced obesity in mouse *in vivo*

**FIGURE 3:**
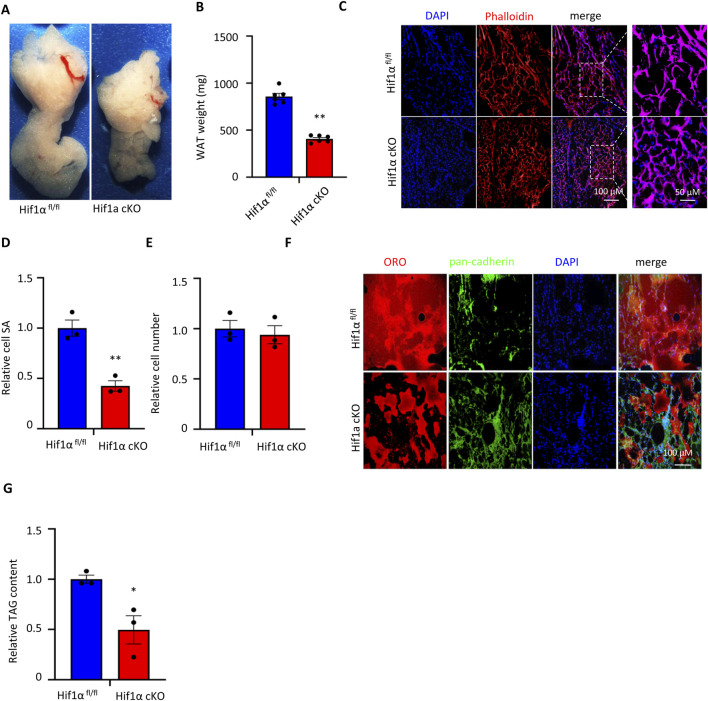
Adipocyte Hif1α inactivation reduces adipocyte cell size in HFD-induced obesity in mouse *in vivo*: **(A)** representative images depicting the gross morphologies of WATs derived from Hif1α^fl/fl^ and Hif1α cKO mice maintained on the HFD protocol. **(B)** Visceral WAT weights of the Hif1α^fl/fl^ and Hif1α cKO mice maintained on HFD. The data are expressed as mean ± SEM (n = 6); *p < 0.05 vs. Hif1α^fl/fl^ HFD by unpaired two-tailed Student’s t-test. **(C)** WAT sections of the Hif1α^fl/f^ and Hif1α cKO mice maintained on NCD were stained with DAPI (blue) and phalloidin to mark actin (red) for immunofluorescence microscopy analysis. Scale bar: 100 μM. A magnified view of the area within the dotted rectangle in the merged panel is shown on the extreme right. Scale bar: 50 μM. **(D)** Quantification of visceral WAT cell SAs of the HFD-fed Hif1α^fl/fl^ and Hif1α cKO mice. The data are expressed as mean ± SEM (n = 6); **p < 0.01 vs. Hif1α^fl/fl^ HFD by unpaired two-tailed Student’s t-test. **(E)** Quantification of the adipocyte cell numbers derived from the visceral WATs of Hif1α^fl/fl^ and Hif1α cKO mice maintained on HFD. The data are expressed as mean ± SEM (n = 5); *p < 0.05 vs. Hif1α^fl/fl^ HFD by unpaired two-tailed Student’s t-test. **(F)** WAT sections of the Hif1α^fl/fl^ and Hif1α cKO mice maintained on NCD were stained with a neutral lipid marker, ORO (red), and DAPI (blue), as well as counterstained with pan-cadherin (green) for immunofluorescence microscopy analysis. Scale bar: 100 μM. **(G)** Quantification of visceral WAT TAG contents of the Hif1α^fl/fl^ and Hif1α cKO mice. The TAG content was normalized to tissue weight. The data are expressed as mean ± SEM (n = 7); **p < 0.05 vs. Hif1α^fl/fl^ HFD by unpaired two-tailed Student’s t-test. NCD, normal chow diet; HFD, high-fat diet; WAT, white adipose tissue; TAG, triacylglyceride.

We speculated that Hif1α in WAT plays a dominant role in obesity development and potentially underlies the reduction in bodyweight detected in the Hif1α cKO mice upon HFD provision (Figure 2H). Thus, we analyzed the WAT contents in the Hif1α^fl/fl^ and Hif1α cKO mice under HFD conditions. As noted, visceral WAT derived from the Hif1α^fl/fl^ mice under HFD was dramatically larger than that derived from the Hif1α cKO mice (Figures 3A,B). To determine whether the reduced adiposity in the Hif1α cKO mice was due to fewer and/or smaller fat cells, we compared the adipocyte cell sizes and numbers in the Hif1α^fl/fl^ and Hif1α cKO mice. As adipocytes are filled with a large intracellular pool of TAG, the cytoplasm and nucleus are restricted to the cellular periphery, allowing the use of phalloidin as a marker of the cellular boundary in immunofluorescence confocal microscopic analysis of the WAT sections to mark actin filaments. The Hif1α cKO adipocytes were 50% smaller than the Hif1α^fl/fl^ control adipocytes but comparable in numbers (Figures 3C–E). Consistent with the difference in cell sizes, the Hif1α cKO adipocytes demonstrated reduced incorporation of the lipid marker ORO and TAG content compared to the Hif1α^fl/fl^ adipose tissue (Figures 3F,G).

Notably, adipocyte-specific deletion of Hif1α reduced adipocyte hypertrophy without altering the adipocyte numbers, indicating that Hif1α is dispensable for adipocyte formation *per se* but is required for stress-induced lipid storage in mature adipocytes engaged in nutrient overload.

### Hif1α regulates differentiated adipocyte growth through the glycerolipid biosynthetic shunt

**FIGURE 4:**
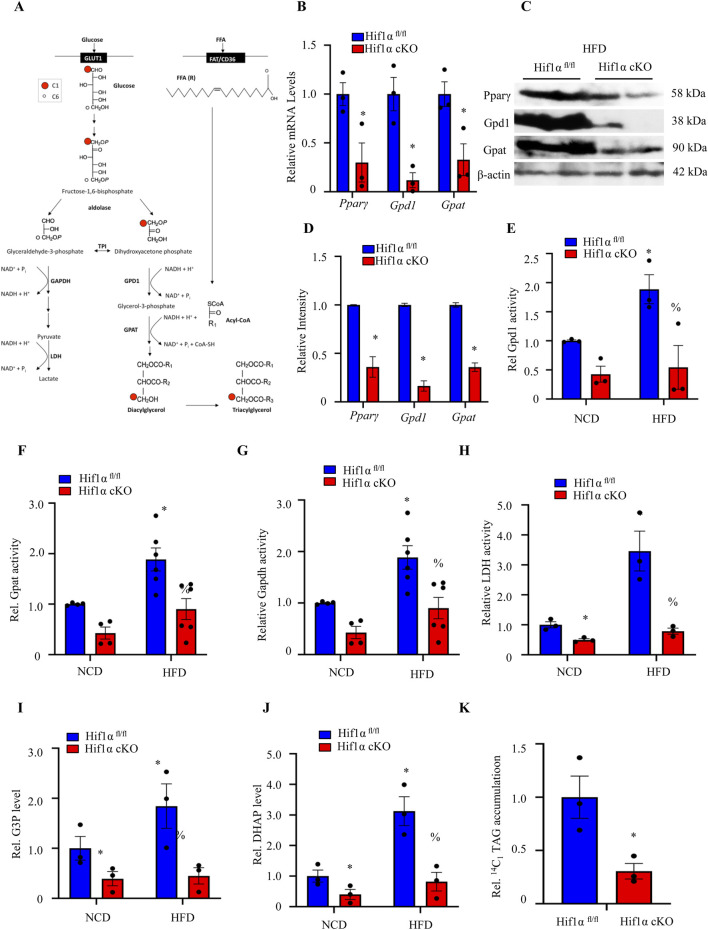
Adipocyte Hif1α inactivation reduces glycerolipid biosynthesis in HFD-induced obesity: **(A)** glycolytic and glycerolipid biosynthesis pathways. **(B)** Relative mRNA expression of *Pparγ*, *Gpd1*, and *Gpat* in the visceral WATs of Hif1α^fl/fl^ and Hif1α cKO mice maintained on the HFD protocol. The data are expressed as mean ± SEM (n = 3); *p < 0.05 vs. Hif1α^fl/fl^ HFD by unpaired two-tailed Student’s t-test. **(C, D)** Western blotting and quantitative densitometry analyses show the protein expression levels of Pparγ, Gpd1, and Gpat in the visceral WATs of Hif1α^fl/fl^ and Hif1α cKO mice maintained on the HFD protocol. **(E)** Visceral WATs of Hif1α^fl/fl^ and Hif1α cKO mice were assessed for endogenous Gpd1 enzymatic activity. The data are expressed as mean ± SEM (n = 3); *p < 0.05 vs. Hif1α^fl/fl^ NCD and ^%^ *p* < 0.05 vs. Hif1α^fl/fl^ HFD by unpaired two-tailed Student’s t-test. **(F)** Visceral WATs of Hif1α^fl/fl^ and Hif1α cKO mice were assessed for endogenous Gpat enzymatic activity. The data are expressed as mean ± SEM (n = 4–6); *p < 0.05 vs. Hif1α^fl/fl^ NCD and ^%^ *p* < 0.05 vs. Hif1α^fl/fl^ HFD by unpaired two-tailed Student’s t-test. **(G)** Visceral WATs of Hif1α^fl/fl^ and Hif1α cKO mice were assessed for endogenous Gpat enzymatic activity. The data are expressed as mean ± SEM (n = 3); *p < 0.05 vs. Hif1α^fl/fl^ NCD and ^%^ *p* < 0.05 vs. Hif1α^fl/fl^ HFD by unpaired two-tailed Student’s t-test. **(H)** Visceral WATs of Hif1α^fl/fl^ and Hif1α cKO mice were assessed for endogenous LDH enzymatic activity. The data are expressed as mean ± SEM (n = 3); *p < 0.05 vs. Hif1α^fl/fl^ NCD and ^%^ *p* < 0.05 vs. Hif1α^fl/fl^ HFD by unpaired two-tailed Student’s t-test. **(I)** G3P level measurements in Hif1α^fl/fl^ and Hif1α cKO mice maintained on either the NCD or HFD protocol. The data are expressed as mean ± SEM (n = 3); *p < 0.05 vs. Hif1α^fl/fl^ NCD and ^%^ *p* < 0.05 vs. Hif1α^fl/fl^ HFD by unpaired two-tailed Student’s t-test. **(J)** DHAP level measurements in Hif1α^fl/fl^ and Hif1α cKO mice maintained on either the NCD or HFD protocol. The data are expressed as mean ± SEM (n = 3); *p < 0.05 vs. Hif1α^fl/fl^ NCD and ^%^ *p* < 0.05 vs. Hif1α^fl/fl^ HFD by unpaired two-tailed Student’s t-test. **(K)** ^14^C1-TAG accumulation measurements in Hif1α^fl/fl^ and Hif1α cKO mice maintained on the HFD protocol. The data are expressed as mean ± SEM (n = 3); *p < 0.05 vs. Hif1α^fl/fl^ HFD by unpaired two-tailed Student’s t-test. Gpd1, glycerol-3-phosphate dehydrogenase 1; Gpat, glycerol-phosphate acyltransferase; Gapdh, glyceraldehyde-3-phosphate dehydrogenase; LDH, lactate dehydrogenase; G3P, glyceraldehyde-3-phosphate; DHAP, dihydroxyacetone phosphate; TAG, triacylglyceride; NCD, normal chow diet; HFD, high-fat diet; WAT, white adipose tissue.

Based on our observations, we investigated if the reduced WAT growth in Hif1α cKO mice maintained on HFD was a result of altered Pparγ activation and reduced flux through the glycerolipid biosynthesis pathway in the adipocytes (Figure 4A). As shown in Figures 4B–D, the RNA and protein expression levels of Pparγ and its gene targets Gpd1 and Gpat were reduced in the Hif1α cKO WAT biopsies compared to controls. Although the lower levels of Gpd1 and Gpat observed in the Hif1α cKO mice maintained on HFD are suggestive of decreased *de novo* TAG synthesis capacity, we directly assessed if the flux through the glycerolipid shunt was altered in the WAT of Hif1α cKO mice. To this end, the WAT biopsies of the Hif1α^fl/fl^ and Hif1α cKO mice maintained on HFD were assessed for Gpd1 and Gpat enzymatic activities (Figures 4E,F). Gpd1 and Gpat are relevant markers of flux through the glycerolipid shunt as Gpd1 is the first step of the glycerolipid biosynthesis pathway and Gpat is the rate-limiting step for *de novo* TAG synthesis. Nutrient overload induced significant increases in the enzymatic activities of the glycerolipid pathway enzymes Gpd1 and Gpat in Hif1α^fl/fl^ WAT (Figures 4E,F). However, inactivation of Hif1α function in the adipocytes prevented induction of Gpd1 and Gpat enzymatic activities in response to nutrient overload (Figures 4E,F). Thus, Hif1α regulates flux through the glycerolipid biosynthetic shunt.

Glycolysis-driven fructose-1,6-bisphosphate breakdown by aldolase produces dihydroxyacetone phosphate (DHAP) and glyceraldehyde-3-phosphate (G3P). This generated DHAP can be either dehydrogenated via the glycerolipid shunt to form glycerol-3-phosphate by Gpd1 or converted to glyceraldehyde-3-phosphate and returned to the glycolytic branch by triose phosphate isomerase (TPI). Accordingly, we reasoned that in the context of increased HIF1α-mediated glycolytic capacity and increased flux into the glycerolipid biosynthetic shunt, HIF1α function could ensure a coordinate engagement of the glycolytic and glycerolipid biosynthetic branches (Figure 4A), thereby promoting the generation of glycolytic intermediates and their channeling into and utilization by the glycerolipid biosynthetic shunt simultaneously. To assess if the establishment of such a mode of coregulation exists in adipocytes, we assayed the enzymatic activity along the canonical Gapdh–Ldh branch. Gapdh activity is a key rate-limiting step in glycolysis, while Ldh serves as a readout for the overall glycolytic rate. As noted in Figures 4G,H, both Gapdh and Ldh activities were markedly reduced in the Hif1α cKO WAT biopsies compared to controls. Accordingly, we detected coordination between the canonical Gapdh–Ldh and Gpd1–Gpat axes in isolated primary adipocytes of the Hif1α^fl/fl^ and Hif1α cKO mice as well as in the lipogenic NIH 3T3-L1 cells (Supplementary Figures S2A–G). These findings not only confirm the coordination of glycolysis with glycerolipid biosynthesis specifically in the adipocytes but further reveal the plasticity of the response to Hif1α function (Supplementary Figures S2A–G).

If the Hif1α function indeed coordinates engagement of the glycolytic and glycerolipid biosynthetic branches simultaneously (by increasing flux from glycolysis for rechanneling toward the glycerolipid biosynthetic branch), we can predict an impact of Hif1α deficiency on the relative levels of the breakdown products of the aldolase reaction (Figure 4A). Hence, we determined if the conversion of fructose-1,6-bisphosphate to either glyceraldehyde-3-phosphate or DHAP (by aldolase) was altered between the Hif1α^fl/fl^ and Hif1α cKO WATs. Biopsies derived from the respective genotypes were first depleted of endogenous fructose-1,6-bisphosphate, glyceraldehyde-3-phosphate, DHAP, and glycerol-3-phosphate. Then, exogenous fructose-1,6-bisphosphate was added, followed by the products of the aldolase reactions; then, glyceraldehyde-3-phosphate and DHAP were measured. Nutrient overloading enhanced the production of glyceraldehyde-3-phosphate and DHAP in Hif1α^fl/fl^ WAT, while Hif1α deletion blocked nutrient-overload-induced metabolite production (Figures 4I,J). Together, these data suggest a role of adipocyte Hif1α in regulating diet-induced adiposity through coregulation of glycolysis and rechanneling of the glycolytic intermediates into the glycerolipid biosynthetic pathway for *de novo* TAG synthesis.

As a result of glucose metabolism, DHAP generated from the breakdown of fructose-1-6-bisphosphate by aldolase retains the carbon moiety at position 1 of glucose (C1), which upon conversion to glycerol-3-phosphate by Gpd1 is acylated by Gpat to culminate in TAG incorporation (Figure 4A). Glucose labeled at C1 is predicted to incorporate into TAG in the adipocytes of the Hif1α^fl/fl^ mice maintained on HFD to a greater extent than the Hif1α cKO adipocytes if Hif1α indeed promotes preferential channeling of the glycolytic intermediate DHAP toward TAG synthesis and if the glycerol component of the stored lipids were derives from the glycolytic cascade. ^14^C1-glucose was added to WAT explants derived from the Hif1α^fl/fl^ and Hif1α cKO mice subjected to the HFD, and the stored TAG was extracted and analyzed for ^14^C1 incorporation. As shown in Figure 4K, ^14^C1 incorporation into TAG was significantly reduced in the Hif1α cKO biopsies compared to controls. Thus, Hif1α activation in response to nutrient overload significantly contributes to the conversion of glucose-derived carbon to lipid synthesis and adipocyte hypertrophy.

## Discussion

**FIGURE 5:**
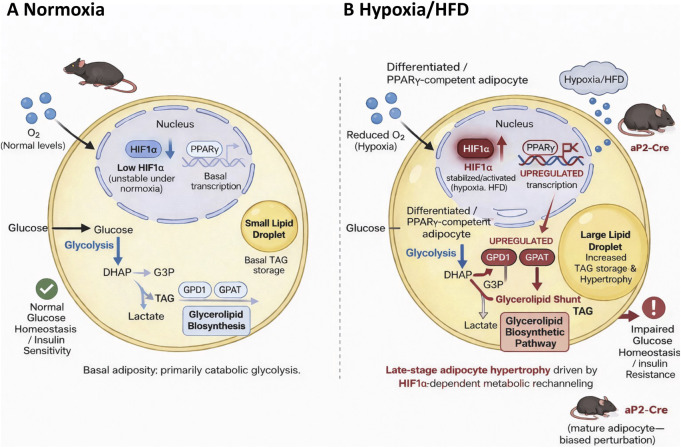
HIF1α executes a late-stage PPARγ-dependent lipid storage program in differentiated adipocytes. The schematic illustrates state-dependent metabolic reprogramming in differentiated adipocytes under normoxic vs. hypoxic or HFD conditions. **(A)** Under normoxia, HIF1α remains unstable and transcriptionally inactive in the differentiated adipocytes. The glycolytic flux primarily supports catabolic metabolism with limited diversion of the glycolytic intermediates into glycerolipid biosynthesis. Basal GPD1 and GPAT activities sustain low-level TAG synthesis, resulting in small lipid droplets as well as glucose homeostasis and insulin sensitivity preservation. **(B)** Under hypoxia or HFD, HIF1α is stabilized and transcriptionally activated in the differentiated PPARγ-competent adipocytes. HIF1α activation is associated with enhanced glycolysis and increased rechanneling of DHAP into the glycerolipid biosynthetic pathway via upregulation of GPD1 and GPAT. This metabolic shunt promotes increased TAG synthesis, lipid droplet expansion, and adipocyte hypertrophy, thereby impairing glucose homeostasis and insulin resistance. Genetic perturbation using aP2-Cre primarily targets mature adipocytes. HFD, high-fat diet; TAG, triacylglyceride; PPARγ, peroxisome proliferator-activated receptor gamma; DHAP, dihydroxyacetone phosphate; GPD1, glycerol-3-phosphate dehydrogenase 1; GPAT, glycerol-3-phosphate acyltransferase.

In the present study, we provide *in vivo* metabolic and enzymatic resolutions for the late-stage prolipogenic arm of HIF1α signaling in adipose tissues. Recent works have established that HIF1α exerts state-dependent functions in adipocytes to inhibit early differentiation and promote lipid accumulation in mature Pparγ-competent cells (Kudo et al., 2023). Building on this framework, the present study is focused specifically on differentiated adipocytes under nutrient excess and hypoxic stress conditions to identify the metabolic pathways through which HIF1α executes these biological programs (Figure 5).

Using adipocyte-specific deletion of Hif1α combined with enzymatic assays and isotope-based metabolic flux analyses, we demonstrate that HIF1α is dispensable for adipocyte formation *per se* but is required for stress-induced lipid accumulation in mature adipocytes. Loss of HIF1α attenuates adipocyte hypertrophy without reducing the adipocyte numbers, consistent with previous reports based on adipocyte-targeted Hif1α perturbation (Jiang et al., 2011; Krishnan et al., 2012) and late-stage perturbation outcomes predicted by the framework described by Kudo et al. (2023). This framework functionally separates adipocyte differentiation from hypertrophy through Pparγ insulation. Within this context, our data identify the Gpd1–Gpat axis as a specific enzymatic driver of the hypertrophic arm of this model. Together, these findings indicate that HIF1α does not regulate adipogenic commitment or lineage determination *in vivo* but instead reinforces lipid storage programs in differentiated adipocytes.

Mechanistically, we identified coordinated regulation of glycolysis and glycerolipid biosynthesis as the central metabolic processes underlying this effect. HIF1α is well established as a transcriptional activator of glycolytic enzymes and glucose transporters (Kim et al., 2006; Gordan et al., 2007), while PPARγ is a master regulator of lipid anabolic gene expression in the adipocytes (Rosen et al., 2002; He et al., 2003). In line with prior work demonstrating integration of the glycolytic and lipid anabolic pathways downstream of the HIF1α–PPARγ axis in pathological cardiac hypertrophy (Krishnan et al., 2009), we show that HIF1α promotes flux through upper glycolysis in adipocytes while simultaneously reinforcing the PPARγ-dependent expression and activities of GPD1 and GPAT as two rate-limiting enzymes in TAG synthesis.

Our enzymatic and tracer-based flux analyses further demonstrate that HIF1α enables efficient partitioning of fructose-1,6-bisphosphate-derived intermediates between canonical glycolysis and the glycerolipid shunt. In this configuration, DHAP generated by aldolase is preferentially converted to glycerol-3-phosphate via GPD1 and subsequently acylated by GPAT to form TAGs. Loss of HIF1α disrupts this coordinated flux, resulting in reduced activities of both glycolytic and glycerolipid biosynthetic enzymes, diminished incorporation of glucose-derived carbon into the TAGs, and attenuation of adipocyte hypertrophy. These findings help identify the GPD1–GPAT axis as a central enzymatic execution module of the late-stage HIF1α–PPARγ lipid storage program.

Importantly, this mechanism does not imply that HIF1α universally promotes adipose tissue growth. However, it reflects a reinforcement function gated by the adipocyte differentiation state and depends on the presence of an established PPARγ transcriptional network. In the absence of adipogenic commitment or during the early stages of adipocyte differentiation, sustained HIF1α activation has been shown to impair adipogenesis, disrupt extracellular matrix remodeling, and limit adipose tissue expandability (Kudo et al., 2023). Thus, the prolipogenic effects of HIF1α described here are not applicable to preadipocytes or during lineage commitment but are restricted to PPARγ-competent adipocytes exposed to nutrient excess conditions.

At the biochemical level, the coordinated regulation of glycolysis and glycerolipid biosynthesis may be further stabilized by redox coupling between the two pathways. GAPDH-mediated glycolysis consumes NAD^+^ and generates NADH, whereas GPD1-driven glycerol-3-phosphate production consumes NADH and regenerates NAD^+^. This reciprocal use of the redox cofactors provides a plausible mechanism by which high flux can be sustained through both pathways simultaneously in hypertrophic adipocytes under hypoxic conditions, thereby favoring continued glycolytic throughput while preventing redox imbalance.

Our findings also help contextualize some earlier observations linking hypoxia with enhanced lipid synthesis in adipose and other metabolic tissues. Although hypoxia-associated lipid accumulation has been recognized for decades, the transcriptional and metabolic mechanisms underlying this response have remained incompletely defined. The present study shows HIF1α-dependent metabolic rechanneling as a key mechanistic link connecting hypoxic signaling with lipid storage in differentiated adipocytes, providing a unified explanation for these observations within a defined cellular state.

Crucially, lipid accumulation within mature adipocytes must be distinguished from adipocyte differentiation, adipose tissue expandability, and overall adipose tissue health. Earlier studies have demonstrated that HIF1α activation simultaneously promotes lipid storage in existing adipocytes while impairing adipogenesis and tissue architecture, ultimately contributing to adipose tissue dysfunction (Krishnan et al., 2012; Kudo et al., 2023). The present work specifically defines the role of HIF1α in hypertrophic lipid storage in PPARγ-competent adipocytes rather than proposing a generalized requirement for HIF1α in adipose tissue development.

When integrated with existing literature, our findings support a model in which HIF1α exerts opposing effects depending on the adipogenic state, timing, and metabolic context. Before or during adipogenic commitment, HIF1α activation constrains differentiation and adipose tissue expandability. In contrast, HIF1α reinforces lipid anabolic programs through metabolic rechanneling in PPARγ-competent adipocytes exposed to nutrient overload, thereby promoting adipocyte hypertrophy and systemic metabolic dysfunction. Within this framework, the present findings extend rather than contradict prior models of adipocyte HIF1α function and establish HIF1α as a state-dependent metabolic regulator rather than a universal driver of adipose tissue growth.

In conclusion, when interpreted in the context of existing literature, our findings extend rather than redefine the role of HIF1α in adipose tissue biology. HIF1α does not control adipose tissue growth globally or resolve a previously unresolved paradox. Instead, consistent with the framework established by Kudo et al. (2023), HIF1α functions as a state-dependent metabolic integrator that reinforces PPARγ-driven lipid anabolic programs in mature adipocytes under nutrient excess conditions. The identification of the GPD1–GPAT axis as the enzymatic executor of this program provides *in vivo* metabolic validation for the late-stage HIF1α–PPARγ model and refines our understanding of the contributions of hypoxic signaling to adipocyte hypertrophy and metabolic diseases.

## Data Availability

The original contributions presented in the study are included in the article/Supplementary Material, and any further inquiries may be directed to the corresponding authors.

## Supplementary material

SUPPLEMENTARY FIGURE S1 Adipocyte-specific Hif1α ablation does not alter the expression of metabolic genes: **(A)** Liver tissues of Hif1α^fl/fl^ and Hif1α cKO mice maintained on the HFD protocol were stained for pan-cadherin (green), oil red O (ORO, red), and DAPI (blue) and analyzed by immunofluorescence confocal microscopy. Scale bar: 200 μM. **(B)** Representative images of the skeletal muscles of Hif1α^fl/fl^ and Hif1α cKO mice maintained on the HFD protocol. Scale bar: 5 μM. HFD, high-fat diet; WAT, white adipose tissue.

SUPPLEMENTARY FIGURE S2 Adipocyte Hif1α inactivation reduces glycerolipid biosynthesis in adipocytes *ex vivo* and *in vitro*: Primary adipocytes were isolated from HFD-fed Hif1α^fl/fl^ and Hif1α cKO mice, and the activities of **(A)** Gpd1, **(B)** Gpat, **(C)** Gapdh, and **(D)** LDH were assessed. The data are expressed as mean ± standard error or the mean (SEM) (n = 5). **p* < 0.05 vs. Hif1α^fl/fl^ HFD. Unpaired two-tailed Student’s t-tests. Lipogenic NIH 3T3-L1 cells were transduced by lentivirus carrying Hif1α shRNA or Hif1α, and ECAR was assessed through **(E)** the Seahorse analyzer, **(F)** Gapdh activity, and **(G)** LDH level. The data are expressed as mean ± SEM (n = 4). **p* < 0.05 vs. ns control. Unpaired two-tailed Student’s t-tests. HFD, high-fat diet; ECAR, extracellular acidification rate; Gpd1, glycerol-3-phosphate dehydrogenase 1; Gpat, glycerol-phosphate acyltransferase; Gapdh, glyceraldehyde-3-phosphate dehydrogenase; LDH, lactate dehydrogenase; DHAP, dihydroxyacetone phosphate; TAG, triacylglyceride; NCD, normal chow diet; HFD, high-fat diet; WAT, white adipose tissue.
