# Supplementary material for: HIF1α reinforces PPARγ-dependent metabolic rechanneling to support lipid accumulation in adipocytes
Source: Front Mol Med. 2026 Feb 23;6:1716464. doi: 10.3389/fmmed.2026.1716464 (PMC12968251; doi:10.3389/fmmed.2026.1716464)
Supplement: Supplementary file 1 [file DataSheet1.pdf]

Supplementary Figure 1

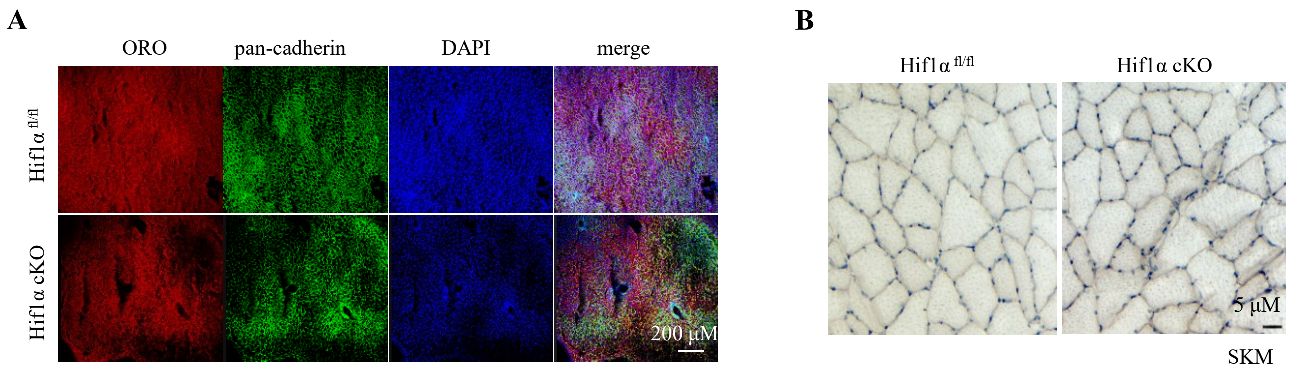

Supplementary Figure 2

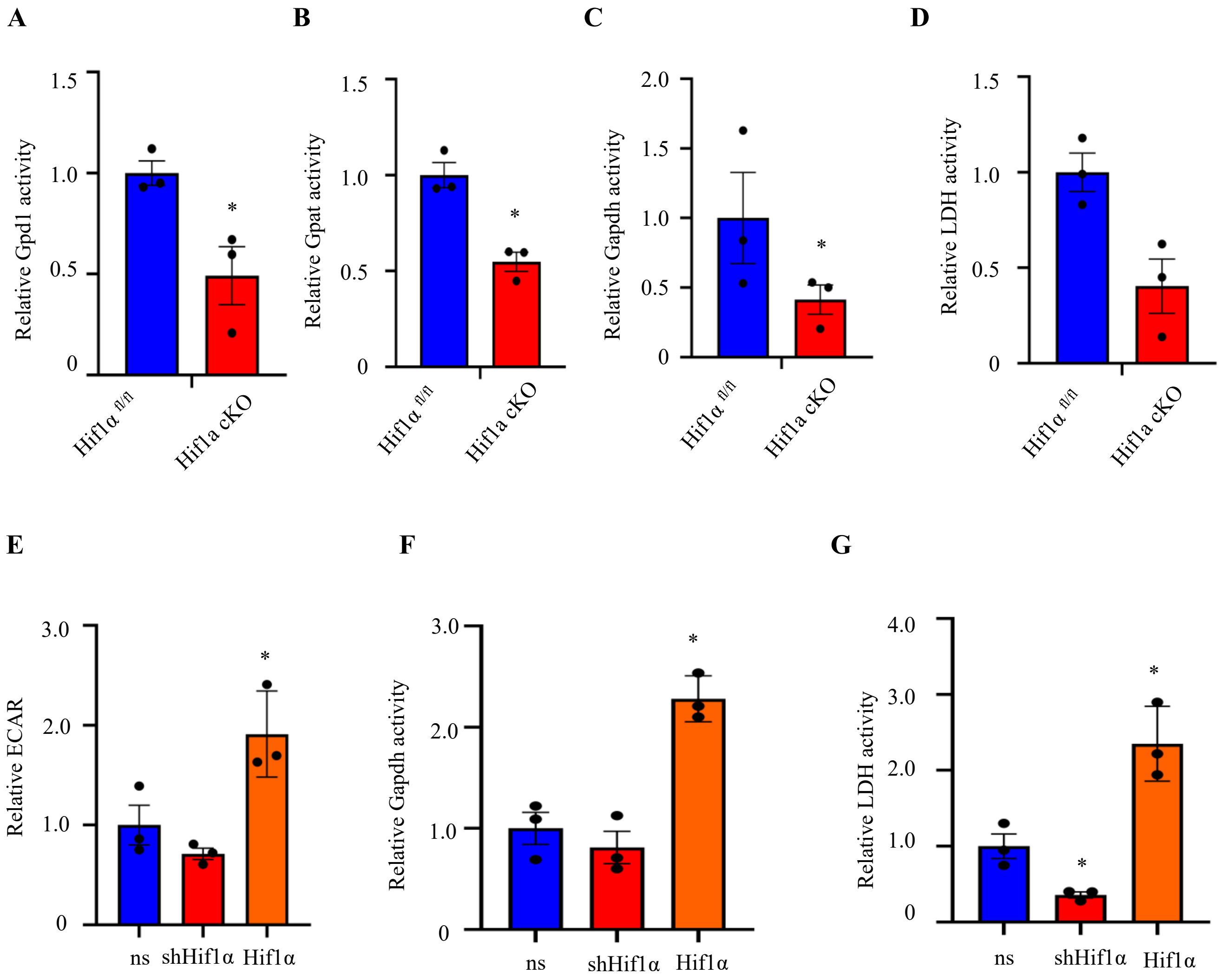

Table 1

Gene	Forward Primer (5'-3')	Reverse Primer (5'-3')
Hif1α	TGCTCATCAGTTGCCACTTC	CGGCATCCAGAAGTTTTCTC
Glut1	GCAGCAAGACCGATGAACAC	CTCCCACAGCCAACATGAGG
Glut4	GGCTCTGACGTAAGGATGGG	GCCACGTTGCATTGTAGCTC
Aldolase A1	CCTTAGTCCTTTCGCCTACCC	GACAGGCGGGTCATGTTGAA
Vegfα	GGAGATCCTTCGAGGAGCACTT	GGCGATTTAGCAGCAGATATAAGAA
Pparγ	CTGCAGGAGCAGAGCAAAG	GAGCAGAGTCACTTGGTCATTC
Gpd1	CTGTCATCGATCCCGACTGG	GGGTAGACAAGTGGCCTGAC
Gpat	TCTCCAGCTTCCAGCTACACA	GGGCTTTGCTTACTGGTCCTG
18S	GTTCGACCATAAACGATGCC	TGGTGGTGCCCTTCCGTCAAT
